# Reduced Cortical Thickness in the Right Caudal Middle Frontal Is Associated With Symptom Severity in Betel Quid-Dependent Chewers

**DOI:** 10.3389/fpsyt.2020.00654

**Published:** 2020-07-10

**Authors:** Adellah Sariah, Weidan Pu, Zhimin Xue, Zhening Liu, Xiaojun Huang

**Affiliations:** ^1^National Clinical Research Centre for Mental Disorders, Institute of Mental Health and Department of Psychiatry, The Second Xiangya Hospital of Central South University, Changsha, China; ^2^Department of Mental Health and Psychiatric Nursing, Hubert Kairuki Memorial University, Dar es Salaam, Tanzania; ^3^Medical Psychological Institute, The Second Xiangya Hospital of Central South University, Changsha, China

**Keywords:** cortical thickness, symptom severity, betel quid dependence, right caudal middle frontal, dorsal lateral prefrontal cortex, decision-making, executive functions, memory

## Abstract

**Background:**

Findings from brain structural imaging studies on betel quid dependence have supported relations between betel quid chewing and alterations in gray matter volume and white matter integrity. However, the effect of betel quid chewing on cortical thickness and the link between cortical thickness and symptom severity remains unascertained.

**Methods:**

In this observational study, we compared cortical thickness measures from 24 male betel quid-dependent chewers with 27 male healthy controls. Using FreeSufer, we obtained three-dimensional T1-weighted images that were used to compute the thickness of the cerebral cortex throughout the cortical layer.

**Results:**

Compared to healthy controls, betel quid dependent chewers displayed significant decreased cortical thickness in the precuneus, entorhinal, right paracentral, middle temporal, and caudal middle frontal gyri. Betel quid dependence scale scores negatively correlated (r = -0.604; *p* = 0.002) with reduced cortical thickness in the right caudal middle frontal of betel quid-dependent chewers.

**Conclusion:**

The findings provide evidence for cortical thickness abnormality in betel dependent chewers and further propose that the severity of betel quid symptoms may be a critical aspect associated with the cortical alterations. The observed alterations may serve as potential mechanisms to explain why BQ chewing behavior is persistent among individuals with betel quid dependence.

## Introduction

Betel quid (BQ) is the fourth frequently used psychoactive substance after caffeine, alcohol, and nicotine (1), consumed by more than 600 million people worldwide (2). Prevalence of BQ dependence (BQD) among users in mainland China ranged from 20.9–33.3% (3), making it a substance of interest in addiction research. BQ consists of four main natural alkaloids including arecoline, arecaidine, guvacoline, and guvacine (4). Arecoline is the main active ingredient (5) assisting the release of dopamine (6) through binding to M5 muscarinic acetylcholine receptors in the ventral tegmental area (VTA) (7). Addictive drugs are thought to increase DA concentration in the major reward network (the VTA, nucleus accumbens, and the prefrontal cortex) (8). Arecoline is also known for its binding properties on GABA receptors in the brain, and influence on smooth muscles, therefore, supporting the reported psychoactive effects (1). For chronic users, BQ has been associated with reduced thinking capacity, mental processing problems, a greater sense of wellness, improved alertness, relaxation, and motor responses right after chewing (9). Some of the pleasant experiences (e.g. arousal, improved alertness, relaxation) have been linked with the observed repetitive BQ chewing behavior that leads to dependence (10). Fresh BQ users on the other hand reported more intense effects than chronic users, suggesting that continual use leads to tolerance (1). Furthermore, individuals with BQ dependence often experience tolerance, craving, BQ seeking behavior, and withdrawal symptoms (3, 11–13) that satisfy the characteristic features for substance abuse (14). Such features stem from continuous exposure of G protein-coupled receptors to arecoline that leads to weaker receptor sensitivity (15), therefore, supporting the occurrence of tolerance and habitual use of substances (10).

Substance dependence is characterized by compulsive intake of drugs, loss of inhibitory control, and negative emotions (such as anxiety, irritability, and dysphoria) portraying withdrawal syndrome in the absence of drugs (16). Previous studies have demonstrated a link between psychoactive substance use and alterations in brain functioning (17–19) and structure (20, 21). Advancement in neuroimaging studies has revealed various approaches used to investigate the effects of psychoactive substances in the brain structure and function of individuals with BQD. Studies have specifically examined the brain function and morphology of BQ dependent chewers by focusing their utmost attention on functional connectivity, white matter integrity, and grey matter (GM) volume alterations. For instance, functional magnetic resonance imaging (fMRI) studies have revealed that betel quid dependence is linked with structural and functional changes in the brain areas responsible for impulsivity, reward, and cognitive processing (22). Specifically, results from BQD resting-state fMRI studies found functional connectivity alterations in orbitofrontal, frontoparietal, occipital/parietal, frontotemporal, medial frontal/anterior cingulate cortex (ACC) networks (23, 24), anterior default mode network (DMN) (23, 25), bilateral precuneus, right insula and right hippocampus (26, 27), temporal and occipital lobe, primary motor cortex (28), ACC-brainstem, and ACC-DMN connectivity (27). Using structural magnetic resonance imaging (sMRI), studies have specifically examined white matter integrity. For example, increased diffusion anisotropy was observed in the right ACC, midbrain, bilateral angular gyrus, right superior temporal gyrus (rSTG), bilateral superior occipital gyrus, left middle occipital gyrus, bilateral superior and inferior parietal lobules, bilateral postcentral, and the precentral gyrus of BQ dependent chewers (29). Furthermore, compared with HC, a higher mean diffusivity and a lower fractional anisotropy were exhibited in anterior thalamic radiation of BQD individuals (30). BQD individuals also demonstrated reduced grey matter (GM) volume in the midbrain, right rostral ACC, bilateral dorsal lateral prefrontal cortex (dlPFC)/insula, and rSTG, while greater GM volume was displayed in the right hippocampal and precuneus (31). Similarly, decreased GM volume was observed in the bilateral ventral medial prefrontal cortex (vmPFC), bilateral dlPFC, and left orbitofrontal cortex (OFC) (32). To date, there is scarcity of studies which have looked into cortical thickness of BQ dependent chewers.

Psychoactive substances are known for their effects on the brain. Progress in neuroimaging research has revealed the roles of various substances in the human brain structure. For example, studies have investigated cortical thickness (CT) abnormalities in individuals dependent on psychoactive substances. For instance, compared with healthy controls (HC), individuals with heroin dependence had decreased CT in the bilateral superior frontal, left caudal middle frontal, right superior temporal, and right insular regions, while increased CT was observed in the left superior parietal, bilateral lingual, left temporal pole, right inferior parietal, right lateral occipital, and right cuneus regions (33). A thinner cortex was observed in the reward regulation and attention function areas of cocaine-dependent patients compared to their matched controls (34). Similarly, compared to controls, alcohol abstainers demonstrated diminished CT in the brain reward system (35), superior frontal, precentral, postcentral, middle frontal, middle/superior temporal, middle temporal, and lateral occipital cortical regions (36). On the other hand, significant decreased CT was observed in the frontal cortex, left caudal ACC, right lateral OFC, left insula, left middle temporal gyrus (MTG), right inferior parietal lobule, and right parahippocampus of young adult smokers (37). Meanwhile, experienced methamphetamine users exhibited thinner CT in the left middle frontal cortex, fronto-polar region, and closeby the central sulcus, than drug naïve controls. However, when compared with low-exposure users, experienced users displayed cortical thinness in the right middle frontal cortex (38). Decreased CT was also displayed in the right caudal middle frontal, bilateral insula, and bilateral superior frontal cortices of adolescent marijuana users compared to controls, whereas the thickness was greater in bilateral lingual, right superior temporal, right inferior parietal, and left paracentral brain regions (39). Additionally, compared to non-cannabis users, reduced CT was observed in the caudal middle frontal gyrus (MFG) of individuals with schizophrenia and bipolar disorder who used cannabis (40). Like other psychoactive substances, exposure to BQ has previously been associated with brain structural anomalies. However, a few studies have examined CT abnormalities in BQ chewers. In one study, individuals with BQD exhibited reduced CT of the dlPFC compared to HC. The observed CT alterations in the dlPFC mediated executive function deficit in BQ dependent individuals (41).

Studies have also advocated the use of symptom severity as an important aspect of understanding the effects of psychoactive drugs on CT abnormalities. For instance, abstinent alcoholics displayed reduced CT which was associated with the severity of alcohol abuse (36). Additionally, the CT of the right dorsal lateral prefrontal cortex (dlPFC) and OFC were associated with the severity of nicotine dependence in young adult smokers (37). Studies have also observed that symptom severity detected by betel quid dependent scale (BQDS) may explain the effects of BQ chewing on brain structural abnormalities (30). Moreover, in a diffusion tensor imaging study, increased mean diffusivity and reduced fractional anisotropy predicted the severity of BQ dependence (30).

Various studies have proposed that dependence on different types of psychoactive substances has similar brain mechanisms (42–44). Basing on this idea, we hypothesized that BQ dependent chewers would display altered CT in some parts of the brain particularly, the caudal MFG (33, 37, 39), paracentral regions (39), and middle temporal cortices (36), of BQ dependent chewers. We also explored if CT in certain parts of the brain could predict symptom severity in BQ dependent chewers. Examining the CT alterations provides further understanding and evidence on the neurobiology of BQ chewing, which explains why individuals with BQD exhibit persistent BQ chewing behavior. Findings from this study may provide possible suggestions for the prevention and treatment of BQD.

## Materials and Methods

### Aim, Design, and Setting of the Study

This observational neuroimaging study examined CT abnormalities and their association with symptom severity in individuals with BQD. Participants’ recruitment and data collection were executed between January 2015 and March 2016 at the Second Xiangya Hospital of Central South University, in Changsha city, Hunan Province, China.

### Characteristics of Participants

Ethical approval for this study was acquired from the Ethics Committee of the Second Xiangya Hospital of Central South University. Before inclusion into the study, each potential participant was asked to provide a signed written informed consent form. The authors had no access to any participant’s identifying information during or after data collection.

The participants of this study were all male. Using the Structured Clinical Interview, participants were assessed to discern if they met the DSM-IV criteria for substance dependence (45). The subsequent inclusion and exclusion have been described before (23, 24, 46). Twenty four individuals with BQD were included in this study. Briefly, participants with BQD had to satisfy the following criteria: 1) 18–40 years of age; 2) Han Chinese ethnicity; 3) completed 9 or more years of education; 4) right-handed; 5) a diagnosis of BQD (a score of ≥5 on the Betel Quid Dependence Scale (BQDS); 6) using BQ at least 1 day at a time for more than 3 years. The BQDS was specifically designed to measure BQD according to the DSM-IV criteria (11). The BQDS is a 16-item self-report instrument encompassing three parts: physical and psychological urgent need, increasing dose, and maladaptive use. The scale has an optimal cut-off score of 4, main sensitivity of 0.926 and specificity of 0.977, and predictive accuracy of up to 99.3%. Furthermore, the BQDS showed high internal consistency (α = 0.921) and exhibited good degrees of validity and reliability for both Chinese and English-speaking BQ chewers (11, 47). Exclusion criteria included: 1) a history of neurological disorder or other serious physical illness; 2) a history of any DSM 5 Axis-I mental disorders; 3) a history of substance abuse other than betel quid; 4) a contraindication to MRI, or 5) a history of electroconvulsive therapy. We recruited 27 HC from the community in Changsha City. The inclusion and exclusion criteria for HC were equivalent to the criteria in the BQD group, except for an additional requirement that; HC did not meet the BQD diagnostic criteria and did not have a family history of psychiatric illness among their first-degree relatives. Individuals with BQD and HC were requested not to use any psychoactive substance in the 24 h preceding scanning. We used Beck Depression (48) and Anxiety Inventories (49) to measure the emotional status of each subject before MRI scanning.

### Images Acquisition

All participants were requested to move as little as possible and foam pads were applied to minimize head motion. Three-dimensional T1-weighted images (3D-T1WIs) were obtained sagittally on a 3.0-Tesla Philips Achieva whole-body MRI scanner (Philips, Netherlands) using turbo field echo sequence with the following parameters: repetition time= 7.5 ms, echo time= 3.7 ms, flip angle=8°, the field of view= 240×240 mm^2^, acquisition matrix= 256×200, slice thickness= 1 mm, gap= 0, number of slices= 180.

### Preprocessing of MRI Data

We carried out a CT analysis of the whole brain through a surface-based approach by employing FreeSurfer tools (version 5.3.0, http://surfer.nmr.harvard.edu) (50, 51). The 3D images were utilized to compute the thickness of the cerebral cortex throughout the cortical layer. In short, the processing stream included a Talairach transform of each of the subject’s native brain, exclusion of non-brain tissue, and separation of grey matter (GM)/white matter (WM) tissue. The GM/WM boundary was tessellated to create multiple vertices across the whole brain. The cortical surface of each hemisphere was enlarged to an average spherical surface to locate the pial surface and the GM/WM boundary. The whole cortex of each subject was visually scrutinized, and topological defects were modified manually, blind to subject identities. CT was measured as the shortest distance between the pial surface and the GM/WM boundary at each vertex across the cortical mantle. The mean CT of each of the 68 regions was calculated using FreeSurfer software (52). The surface maps were smoothed on the normalized CT through a Gaussian kernel with a full-width-half-maximum (FWHM) of 10 mm.

### Statistical Analyses

Statistical Package for the Social Sciences 19.0 (IBM SPSS Inc., USA) was used to compare demographic and clinical characteristics between the two groups. An independent two-sample t-test was used to compare age, years of education, total intracranial volume (TIV), Beck depression, and Beck anxiety inventory scores. A P-value of <0.05 was considered statistically significant.

FreeSurfer’s QDEC 1.4 (query, design, estimate, contrast) application was employed to create statistical maps. QDEC uses the Desikan-Killiany cortical atlas that parcellates the cerebral cortex into 34 cortical regions in each hemisphere (52). It fits a general linear model (GLM) at each surface vertex to describe the data from all participants in the study. Age and education are known to impact brain structure and function (53, 54). Additionally, TIV is also known for its impact on brain structure analysis (55). Basing on that knowledge, these three variables were accounted for as covariates, and regional CT differences were compared between BQD and HC. Using the CT maps, statistical difference maps were created based on the GLM. A Monte Carlo simulation analysis with 10,000 repetitions was performed to correct for multiple comparisons at a threshold of *p*<0.05 with a cluster size of at least 20 voxels.

### Correlation Analysis

Pearson’s correlation was calculated to examine the relationship between the mean values of CT and BQDS scores. Using SPSS 19.0 (*p*<0.05), correlation analysis was implemented to the mean value of the regions showing significantly altered CT between BQ dependent users and HC and BQDS scores.

## Results

### Demographics and Clinical Characteristics

Fifty-one male participants (24 BQD and 27 HC) were involved in the present study. A significant difference in years of education (15.13±1.73 for individuals with BQD; 16.00±0.00 for HC; p=0.01) was observed between individuals with BQD and HC. No significant differences in age and TIV were detected between the groups. Individuals with BQD showed that they had been chewing BQ for a mean duration of 7.75±4.28 years, range 3.25 to 18 years. The average score of BQDS in the BQ dependent group was 7.42±1.86. Individuals with BQD had significantly higher Beck Depression Inventory (BDI) and Beck Anxiety Inventory (BAI) scores than the HC. However, the scores on the BDI (10.58±6.69, range 0 to 24 for the BQ dependent group; 3.89±4.63, range 0 to 14 for the HC group) and BAI (28.50±6.20, range 21 to 45 for the BQ dependent group; 23.19±2.66, range 21 to 32 for the HC group) showed that none of the participants were found with depression or anxiety symptoms. The demographics and clinical characteristics of BQ dependent and HC participants are summarized in **Table 1**.

**Table 1:** Demographics and clinical characteristics of individuals with betel quid dependence (BQD) and healthy controls (HC).

	BQD (Mean ± SD)	HC (Mean ± SD)	*t*/χ^2^	*P*-Value
Age (years)	23.54 (3.87)	24.52 (1.45)	-1.22^a^	0.23
Gender (male/female)	24/0	27/0		
Education (years)	15.13 (1.73)	16.00 (0.00)	-2.64^a^	0.01*
Total Intracranial Volume (cm^3^)	1104.76 (103.7)	1209.59 (229.4)	-2.058	0.05
Betel Quid Dependence Scale	7.42 (1.86)	N/A		
Duration of Betel Quid (years)	7.75 (4.28)	N/A		
Beck Depression Inventory	10.58 (6.69)	3.89 (4.63)	4.20^a^	0.00*
Beck Anxiety Inventory	28.50 (6.20)	23.19 (2.66)	4.06^a^	0.00*

SD, standard deviation; N/A, not applicable; BQD, betel quid dependence; HC, healthy controls; a, independent-samples t-test; *P < 0.05.

### Cortical Thickness Differences Between Individuals With BQD and HC

**Figure 1:**
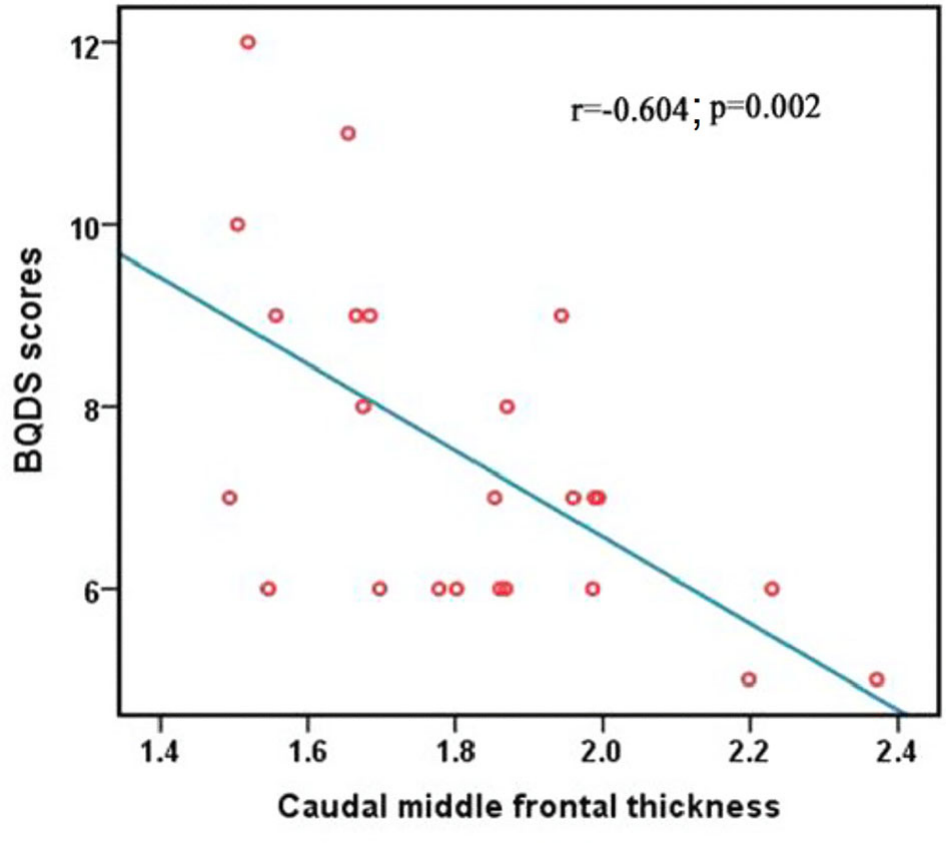
Differences in CT between individuals with BQD and HC. Regions with decreased CT in BQ dependent individuals include the left precuneus, entorhinal, right paracentral, middle temporal gyrus, and caudal middle frontal. The color bars represent the range of t value. L, left; R, right; CT, cortical thickness; BQD, betel quid dependence; HC, healthy control.

Compared with HC, individuals with BQD exhibited decreased CT in left precuneus, left entorhinal, right paracentral, middle temporal, and caudal middle frontal brain regions (two-sample t-test, p<0.05, FDR corrected), (**Table 2**), and (**Figure 1**). Compared to the HC group, no brain regions were found with increased CT in the BQD group.

**Table 2:** Brain regions showing group differences in CT (*p* < 0.05, FDR corrected).

Brain region	Side	Cluster size (voxels)	MNI coordinates			Peak T value
			X	Y	Z	
BQD<HC						
Precuneus	Left	181	-16	-43	65	-4.27
Entorhinal	Left	49	-26	2	-35	-3.22
Paracentral	Right	71	17	-44	47	-3.33
Middle temporal	Right	38	58	-57	6	-2.96
Caudal middle frontal	Right	21	34	5	55	-2.95
BQD>HC						
Negative	–	–			–	–

BQD, betel quid dependence; HC, healthy control; CT, cortical thickness; MNI, Montreal Neurological Institute.

### Correlation Analysis

**Figure 2:**
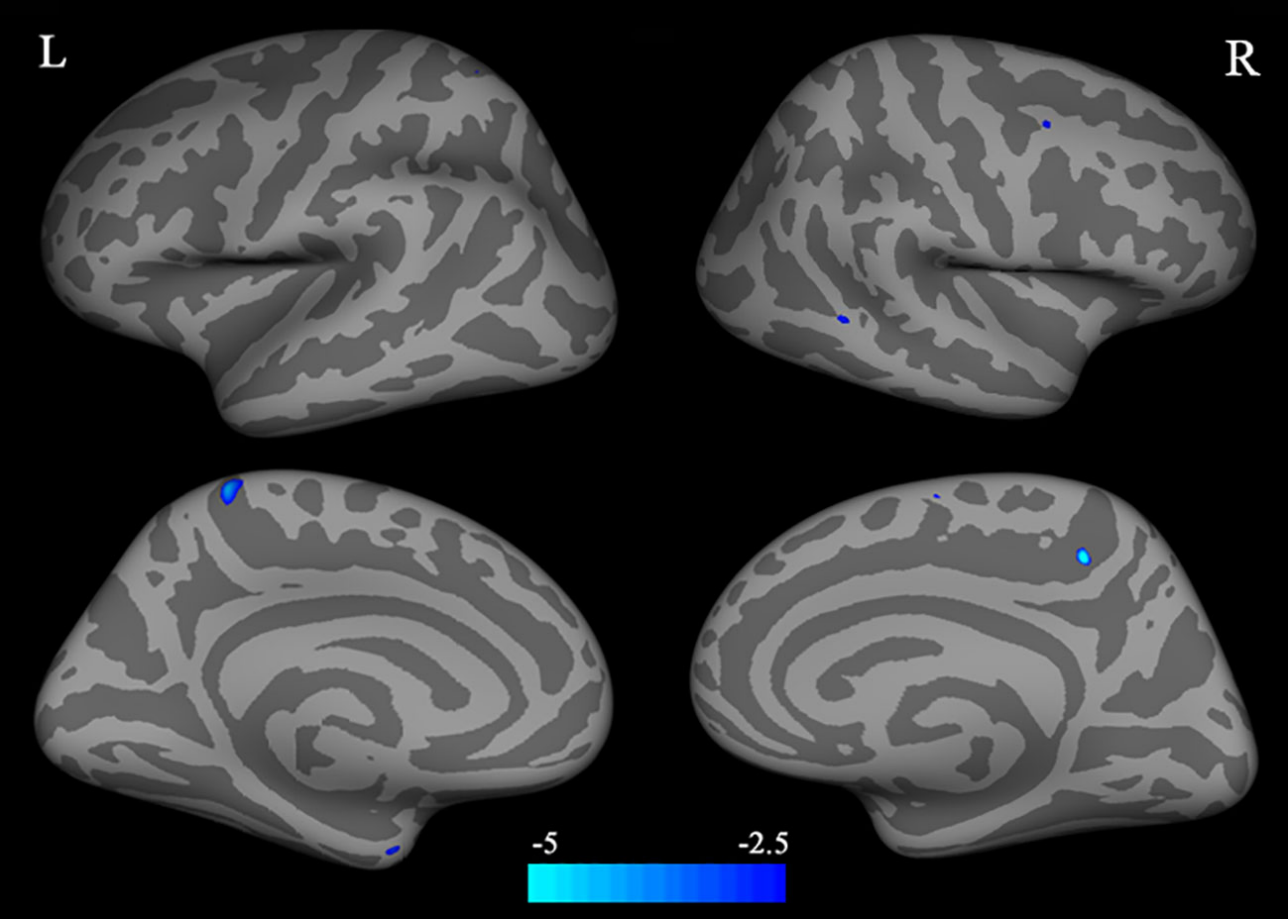
Correlation between BQDS scores and the mean values of CT of the right caudal middle frontal. BQDS, Betel Quid Dependence Scale; CT, cortical thickness.

The BQDS scores negatively correlated with the decreased mean value of CT in the right caudal middle frontal in the BQD group (r = -0.604; *p* = 0.002), (**Figure 2**). The correlation results of the mean value of all regions showing significantly altered CT between the BQD users and HC and BQDS scores are displayed in **Table 3**.

**Table 3:** Pearson’s correlation coefficients between the mean value of cortical thickness (CT) and betel quid dependence scale (BQDS) scores in betel quid dependent individuals (n=24, *p < 0.05).

Cortical thickness	Betel Quid Dependence Scale scores	
	r	p-value
Precuneus (left)	0.341	0.103
Entorhinal (left)	-0.103	0.632
Paracentral (right)	0.032	0.881
Middle temporal (right)	0.121	0.572
Caudal middle frontal (right)	-0.604	0.002*

## Discussion

The hypothesis in our study was supported and we found significant cortical thinning in several areas of the brain. An important finding in the current study is that, compared to HC, individuals with BQD demonstrated reduced CT in the right caudal middle frontal gyrus (MFG) which negatively correlated with symptom severity.

Our results are consistent with findings of a cortical reduction in the caudal MFG among cannabis users (39, 40), and heroin-dependent individuals (33). The MFG (encompassed in the dlPFC) located in the PFC, is known for its role in reorienting attention (56). Dysfunctions in the PFC have been linked with drug compulsivity, craving, denial of illness, and lack of motivation to seek treatment for drug addiction (42). A very recent study found that individuals with BQD displayed reduced CT of the dlPFC compared to HC (41). Moreover, compared with low-urge BQ dependent individuals, high-urge chewers displayed greater activation in areas of the brain concerned with cravings, such as the ACC, medial orbital frontal gyrus, and superior frontal gyrus (57). The dlPFC interacts with various cortical structures and has widely been mentioned in addiction studies. For example, the dlPFC assesses and joins emotional related information from the amygdala and nucleus accumbens and interacts with the dorsal ACC to exert top-down control and metacognitive functions (42). The dlPFC also participates in memory processing (58, 59), executive functions (60), and decision making (61, 62). A previous study has reported that CT of the dlPFC participated in mediating executive function deficits in individuals with BQD (41). The dlPFC has also been linked with cue-induced craving in substance use disorders (63) emanating from drug-related memories (42) that influence reward-related decision making (64). Additionally, the dlPFC is known for its role in conflict resolution. For instance, the dlPFC was associated with improved cognitive control and better ability to resolve conflicts among individuals who had resumed smoking (65). Similarly, methamphetamine-dependent subjects displayed reduced activation in the right middle frontal cortex when presented with conflict trials as a measure of behavior regulation (66). We argue that reduced CT in the right caudal MFG within the dlPFC may compromise decision-making abilities, enhances reward-related memories, leading to a craving, and compulsive BQ chewing behavior. Previous studies have also shown that longer duration of BQ chewing significantly correlated with a reduction in GM volume in the MFG, such that compared with HC, reduced GM volume (31) and short-range functional connectivity (27) were displayed in the dlPFC of BQ dependent individuals.

Our results demonstrated decreased CT in the entorhinal cortex of BQ dependent chewers. Inconsistent with our findings is a study that found marijuana adolescent users with increased CT in the entorhinal cortex (67), suggesting a different neuro-mechanism exerted by different types of psychoactive substances (8). The entorhinal cortex embedded in the anterior medial temporal lobe (68) has numerous projections to the hippocampus and neocortical regions (69) and is presumed to play an important role in learning and memory (70). Drugs of abuse can seize ordinary learning and memory structurethrough pharmacological mechanisms on numerous neurotransmitter systems (71). The mechanism of reward-related learning and memory in addiction is represented by pathologies in gene and protein expression alterations, changes in neuronal activity, and morphological alterations in the neural circuits (72). Addictive drugs can create artificial learning signals that are stronger and last longer than what is normally seen neurochemically as a result of natural events. Positive experience from the substance leads to enhanced learning that results in more repetitive behavior (73), typical of what is demonstrated in BQ dependent chewers.

Although other studies have reported increased CT in the precuneus (74), our study found a significant decrease. The precuneus is known for its involvement in visual imagery, attention, and memory retrievals. It is also linked with visual processing and integrating related memory (75). We argue that reduced CT observed in the precuneus of BQ dependent individuals may be associated with neurodegeneration emanating from chronic BQ chewing. We also observed reduced CT in the right paracentral lobule. Similar to our findings, more alcohol consumption was associated with a thinner paracentral lobule (67). Conversely, increased paracentral CT has been reported in adolescent marijuana users (39), signifying that different addictive substances may exhibit diverse mechanisms in the brain (8).

Consistent with previous studies that examined adult smokers (37), and abstinent alcoholics (36), our study found significant cortical thinning in the middle temporal gyrus. The decreased degree of centrality in the middle temporal gyrus has been associated with language expression (76), attention, and cognitive deficits in addicts (77, 78), suggesting a weakening of cognitive control in individuals with BQD.

There are several limitations to this study. First, the use of a small sample size consisting of only male participants may have influenced the interpretation of our results. Future studies with larger samples comprising male and female subjects may provide robust results. Second, although our observations show that CT abnormalities are associated with BQD, being a cross-sectional study, we cannot infer a cause and effect relationship between CT alterations and BQD. Therefore, future longitudinal neuroimaging studies may be more appropriate to establish the cause and effect phenomenon. Third, nicotine and alcohol are substances commonly used by most Chinese men (79, 80). Even though individuals with alcohol and nicotine abuse were excluded from this study, the influence of such substances in the observed cortical alterations cannot be ascertained. To overcome the possibility of confounding results, it would be useful to design future studies that will examine the potential differences in CT abnormalities between subgroups of individuals with BQD defined by their concomitant use of alcohol and other substances (21).

## Conclusion

Our results provide further compelling evidence for CT abnormalities in BQ dependent chewers and propose that symptom severity is a critical factor linked with brain morphological alterations. CT loss in the right caudal MFG is a significant biological correlate for symptom severity in BQ dependent chewers. We presume that the caudal MFG participates in retrieving BQ reward-related memories which in turn influences one’s decision making about whether to continue chewing BQ, leading to the perpetuation of BQ dependent behavior. However, the observed CT alterations may have preceded BQD and therefore act as risk factors for BQ use and BQD or they may have stemmed from chronic BQ use. Future longitudinal BQ neuroimaging studies may be useful to elucidate these conclusions.

## Data Availability Statement

The raw data supporting the conclusions of this article will be made available by the authors, without undue reservation, to any qualified researcher.

## Ethics Statement

The studies involving human participants were reviewed and approved by Ethics Committee of the Second Xiangya Hospital of Central South University. The patients/participants provided their written informed consent to participate in this study.

## Author Contributions

XH designed the study, collected and analyzed the data. AS prepared the first draft of the manuscript. AS and XH actively participated in writing and revising the manuscript for publication. All authors contributed to the article and approved the submitted version.

## Funding

This study was supported by the China Precision Medicine Initiative (2016YFC0906300) and the National Natural Science Foundation of China (Grant nos.81561168021 and 81671335).

## Conflict of Interest

The authors declare that the research was conducted in the absence of any commercial or financial relationships that could be construed as a potential conflict of interest.

## Abbreviations

CT, Cortical thickness; BQ, Betel quid; BQD, Betel quid dependence; HC, Healthy controls; GM, Grey matter; rSTG, Right superior temporal gyrus; dlPFC, Dorsolateral prefrontal cortex; vmPFC, Ventral medial prefrontal cortex; OFC, Orbital frontal cortex; MTG, Middle temporal gyrus; MFG, Middle frontal gyrus; BQDS, Betel quid dependence scale; WM, White matter.

## References

[1] Boucher BJ Mannan N Metabolic effects of the consumption of Areca catechu. Addict Biol (2002) 7(1):103–10. 10.1080/13556210120091464 11900629

[2] Gupta PC Warnakulasuriya S Global epidemiology of areca nut usage. Addict Biol (2002) 7(1):77–83. 10.1080/13556210020091437 11900626

[3] Lee C-H Ko AM-S Yen C-F Chu K-S Gao Y-J Warnakulasuriya S Betel-quid dependence and oral potentially malignant disorders in six Asian countries. Br J Psychiatry (2012) 201(05):383–91. 10.1192/bjp.bp.111.107961 22995631

[4] Franke AA Mendez AJ Lai JF Arat-Cabading C Li X Custer LJ Composition of betel specific chemicals in saliva during betel chewing for the identification of biomarkers. Food Chem Toxicol (2015) 80:241–6. 10.1016/j.fct.2015.03.012 PMC4454402 25797484

[5] Chu N-S Effects of betel chewing on the central and autonomic nervous systems. J Biomed Sci (2001) 8(3):229–36. 10.1007/BF02256596 11385294

[6] Bendor J Lizardi-Ortiz JE Westphalen RI Brandstetter M Hemmings HC Sulzer D AGAP1/AP-3-dependent endocytic recycling of M5 muscarinic receptors promotes dopamine release. EMBO J (2010) 29(16):2813–26. 10.1038/emboj.2010.154 PMC2924642 20664521

[7] Yang K Buhlman L Khan GM Nichols RA Jin G McIntosh JM Functional Nicotinic Acetylcholine Receptors Containing 6 Subunits Are on GABAergic Neuronal Boutons Adherent to Ventral Tegmental Area Dopamine Neurons. J Neurosci (2011) 31(7):2537–48. 10.1523/JNEUROSCI.3003-10.2011 PMC3081713 21325521

[8] Sulzer D How Addictive Drugs Disrupt Presynaptic Dopamine Neurotransmission. Neuron (2011) 69(4):628–49. 10.1016/j.neuron.2011.02.010 PMC3065181 21338876

[9] Osborne P Chou T-S Shen T-W Characterization of the Psychological, Physiological, psychological and EEG profiles of Acute Betel Quid Intoxication naive subjects. PloS One (2011) 6(8):1–11. 10.1371/journal.pone.0023874 PMC3166120 21909371

[10] Osborne P Ko Y-C Wu M-T Lee C-H Intoxication and substance use disorder to Areca catechu nut containing betel quid: A review of epidemiological evidence, pharmacological basis and social factors influencing quitting strategies. Drug Alcohol Depend (2017) 179:187–97. 10.1016/j.drugalcdep.2017.06.039 28787696

[11] Lee C-Y Chang C-S Shieh T-Y Chang Y-Y Development and validation of a self-rating scale for betel quid chewers based on a male-prisoner population in Taiwan: The Betel Quid Dependence Scale. Drug Alcohol Depend (2012) 121(1–2):18–22. 10.1016/j.drugalcdep.2011.07.027 21955360

[12] Bhat SJS Blank MD Balster RL Nichter M Nichter M Areca nut dependence among chewers in a South Indian community who do not also use tobacco: Areca nut topography and dependence. Addiction (2010) 105(7):1303–10. 10.1111/j.1360-0443.2010.02952.x PMC3143027 20642513

[13] Lee C-H Chiang S-L Ko AM-S Hua C-H Tsai M-H Warnakulasuriya S Betel-quid dependence domains and syndrome associated with betel-quid ingredients among chewers: an Asian multi-country evidence: Betel-quid dependence and betel-quid ingredients. Addiction (2014) 109(7):1194–204. 10.1111/add.12530 24650227

[14] Garg A Chaturvedi P Mishra A Datta S A review on harmful effects of pan masala. Indian J Cancer (2015) 52(4):663. 10.4103/0019-509X.178449 26960511

[15] Wess J Eglen RM Gautam D Muscarinic acetylcholine receptors: mutant mice provide new insights for drug development. Nat Rev Drug Discov (2007) 6:721. 10.1038/nrd2379 17762886

[16] Goldstein RZ Volkow ND Drug Addiction and Its Underlying Neurobiological Basis: Neuroimaging Evidence for the Involvement of the Frontal Cortex. Am J Psychiatry (2002) 159(10):1642–52. 10.1176/appi.ajp.159.10.1642 PMC1201373 12359667

[17] Khalili-Mahani N Zoethout RMW Beckmann CF Baerends E de Kam ML Soeter RP Effects of morphine and alcohol on functional brain connectivity during “resting state”:A placebo-controlled crossover study in healthy young men. Hum Brain Mapp (2012) 33(5):1003–18. 10.1002/hbm.21265 PMC6870105 21391283

[18] Konova AB Moeller SJ Tomasi D Volkow ND Goldstein RZ Effects of Methylphenidate on Resting-State Functional Connectivity of the Mesocorticolimbic Dopamine Pathways in Cocaine Addiction. JAMA Psychiatry (2013) 70(8):857. 10.1001/jamapsychiatry.2013.1129 23803700 PMC4358734

[19] Ma N Liu Y Li N Wang C-X Zhang H Jiang X-F Addiction related alteration in resting-state brain connectivity. NeuroImage (2010) 49(1):738–44. 10.1016/j.neuroimage.2009.08.037 PMC2764798 19703568

[20] Daumann J Koester P Becker B Wagner D Imperati D Gouzoulis-Mayfrank E Medial prefrontal gray matter volume reductions in users of amphetamine-type stimulants revealed by combined tract-based spatial statistics and voxel-based morphometry. NeuroImage (2011) 54(2):794–801. 10.1016/j.neuroimage.2010.08.065 20817105

[21] Ersche KD Barnes A Jones PS Morein-Zamir S Robbins TW Bullmore ET Abnormal structure of frontostriatal brain systems is associated with aspects of impulsivity and compulsivity in cocaine dependence. Brain (2011) 134(7):2013–24. 10.1093/brain/awr138 PMC3122375 21690575

[22] Sariah A Liu Z Pu W Liu H Xue Z Huang X Structural and Functional Alterations in Betel-Quid Chewers: A Systematic Review of Neuroimaging Findings. Front Psychiatry (2019) 10:16. 10.3389/fpsyt.2019.00016 30761025 PMC6361845

[23] Huang X Liu Z Mwansisya TE Pu W Zhou L Liu C Betel quid chewing alters functional connectivity in frontal and default networks: A resting-state fMRI study: Betel Quid Alters Network Connectivity. J Magn Reson Imaging (2016) 45(1):157–66. 10.1002/jmri.25322 27227967

[24] Huang X Pu W Liu H Li X Greenshaw AJ Dursun SM Altered Brain Functional Connectivity in Betel Quid-Dependent Chewers. Front Psychiatry (2017) 8:1–9. 10.3389/fpsyt.2017.00239/full 29209234 PMC5701933

[25] Zhu X Zhu Q Jiang C Shen H Wang F Liao W Disrupted Resting-State Default Mode Network in Betel Quid-Dependent Individuals. Front Psychol (2017) 8:1–9. 10.3389/fpsyg.2017.00084/full 28194128 PMC5276995

[26] Weng J-C Chou Y-S Huang G-J Tyan Y-S Ho M-C Mapping brain functional alterations in betel-quid chewers using resting-state fMRI and network analysis. Psychopharmacology (2018) 1–15. 10.1007/s00213-018-4841-8 29441422

[27] Liu T Li J Zhang Z Xu Q Lu G Huang S Altered Long- and Short-Range Functional Connectivity in Patients with Betel Quid Dependence: A Resting-State Functional MRI Study. Cell Physiol Biochem (2016) 40(6):1626–36. 10.1159/000453212 28006783

[28] Liu T Li J Zhao Z Yang G Pan M Li C Altered Spontaneous Brain Activity in Betel Quid Dependence: A Resting-state Functional Magnetic Resonance Imaging Study. Medicine (2016) Feb 95(5):e2638. 10.1097/MD.0000000000002638 PMC4748897 26844480

[29] Weng J-C Kao T-W Huang G-J Tyan Y-S Tseng H-C Ho M-C Evaluation of structural connectivity changes in betel-quid chewers using generalized q-sampling MRI. Psychopharmacology (2017) Jul 234(13):1945–55. 10.1007/s00213-017-4602-0 28342092

[30] Yuan F Zhu X Kong L Shen H Liao W Jiang C White Matter Integrity Deficit Associated with Betel Quid Dependence. Front Psychiatry (2017) 8:1–6. 10.3389/fpsyt.2017.00201/full 29075207 PMC5643420

[31] Chen F Zhong Y Zhang Z Xu Q Liu T Pan M Gray matter abnormalities associated with betel quid dependence: A voxel-based morphometry study. Am J Trans Res (2015) 7(2):364–74. PMC4399099 25901203

[32] Yuan F Kong L Zhu X Jiang C Fang C Liao W Altered Gray-Matter Volumes Associated With Betel Quid Dependence. Front Psychiatry (2017) 8:1–6. 10.3389/fpsyt.2017.00139/full 28824470 PMC5540953

[33] Li M Tian J Zhang R Qiu Y Wen X Ma X Abnormal cortical thickness in heroin-dependent individuals. NeuroImage (2014) 88:295–307. 10.1016/j.neuroimage.2013.10.021 24140937

[34] Makris N Gasic GP Kennedy DN Hodge SM Kaiser JR Lee MJ Cortical Thickness Abnormalities in Cocaine Addiction—A Reflection of Both Drug Use and a Pre-existing Disposition to Drug Abuse? Neuron (2008) 60(1):174–88. 10.1016/j.neuron.2008.08.011 PMC3772717 18940597

[35] Durazzo TC Tosun D Buckley S Gazdzinski S Mon A Fryer SL Cortical Thickness, Surface Area, and Volume of the Brain Reward System in Alcohol Dependence: Relationships to Relapse and Extended Abstinence: Brain Reward System In Relapse And Abstinence. Alcohol: Clin Exp Res (2011) 35(6):1187–200. 10.1111/j.1530-0277.2011.01452.x PMC3097308 21410483

[36] Fortier CB Leritz EC Salat DH Venne JR Maksimovskiy AL Williams V Reduced Cortical Thickness in Abstinent Alcoholics and Association with Alcoholic Behavior: Reduced Cortical Thickness In Abstinent Alcoholics. Alcohol: Clin Exp Res (2011) 35(12):2193–201. 10.1111/j.1530-0277.2011.01576.x PMC3613762 21919920

[37] Li Y Yuan K Cai C Feng D Yin J Bi Y Reduced frontal cortical thickness and increased caudate volume within fronto-striatal circuits in young adult smokers. Drug Alcohol Depend (2015) 151:211–9. 10.1016/j.drugalcdep.2015.03.023 25865908

[38] Koester P Tittgemeyer M Wagner D Becker B Gouzoulis-Mayfrank E Daumann J Cortical thinning in amphetamine-type stimulant users. Neuroscience (2012) 221:182–92. 10.1016/j.neuroscience.2012.06.049 22750208

[39] Lopez-Larson MP Bogorodzki P Rogowska J McGlade E King JB Terry J Altered prefrontal and insular cortical thickness in adolescent marijuana users. Behav Brain Res (2011) 220(1):164–72. 10.1016/j.bbr.2011.02.001 PMC3073407 21310189

[40] Hartberg CB Lange EH Lagerberg TV Haukvik UK Andreassen OA Melle I Cortical thickness, cortical surface area and subcortical volumes in schizophrenia and bipolar disorder patients with cannabis use. Eur Neuropsychopharmacol (2018) 28(1):37–47. 10.1016/j.euroneuro.2017.11.019 29254657

[41] Zhu X Liu S Liao W Kong L Jiang C Yuan F Executive function deficit in betel-quid-dependent chewers: Mediating role of prefrontal cortical thickness. J Psychopharmacol (2018) 32(12):1362–8. 10.1177/0269881118806299 30379118

[42] Goldstein RZ Volkow ND Dysfunction of the prefrontal cortex in addiction: neuroimaging findings and clinical implications. Nat Rev Neurosci (2011) 12(11):652–69. 10.1038/nrn3119 PMC3462342 22011681

[43] Volkow ND Fowler JS Addiction, a diseaseof compulsion and drive: Involvement of the orbital frontal cortex. Cereb Cortex (2000) 10(3):318–25. 10.1093/cercor/10.3.318 10731226

[44] Volkow ND Wang G-J Fowler JS Tomasi D Addiction circuitry in the human brain. Annu Rev Pharmacol Toxicol (2012) 52:321–36. 10.1146/annurev-pharmtox-010611-134625 PMC3477468 21961707

[45] Spitzer RL Williams JBW Gibbon M First MB The Structured Clinical Interview for DSM-III-R (SCID)_History, rationale and description. Arch Gen Psychiatry (1992) 49:624–6. 10.1001/archpsyc.1992.01820080032005 1637252

[46] Sariah A Guo S Zuo J Pu W Liu H Rolls ET Acute and Chronic Effects of Betel Quid Chewing on Brain Functional Connectivity. Front Psychiatry (2020) 11:198. 10.3389/fpsyt.2020.00198 32256411 PMC7094756

[47] Herzog TA Murphy KL Little MA Suguitan GS Pokhrel P Kawamoto CT The Betel Quid Dependence Scale: Replication and extension in a Guamanian sample. Drug Alcohol Depend (2014) 138:154–60. 10.1016/j.drugalcdep.2014.02.022 PMC4010585 24629627

[48] Beck AT Steerer RA Manual for the Beck Depression Inventory-II. San Antonio, TX: Psychological Corporation (1996).

[49] Beck AT Epstein N Brown G Steerer RA An inventory for measuring clinical anxiety: Psychometric properties. J Consult Clin Psychol (1988) 56:893–7. 10.1037/0022-006X.56.6.893 3204199

[50] Dale AM Fischl B Sereno MI Cortical Surface-Based Analysis. NeuroImage (1999) 9:179–94. 10.1006/nimg.1998.0395 9931268

[51] Fischl B Sereno MI Dale AM Cortical Surface-Based Analysis. NeuroImage (1999) 9:195–207. 10.1006/nimg.1998.0396 9931269

[52] Desikan RS Ségonne F Fischl B Quinn BT Dickerson BC Blacker D An automated labeling system for subdividing the human cerebral cortex on MRI scans into gyral based regions of interest. NeuroImage (2006) 31(3):968–80. 10.1016/j.neuroimage.2006.01.021 16530430

[53] Carne RP Vogrin S Litewka L Cook MJ Cerebral cortex: An MRI-based study of volume and variance with age and sex. J Clin Neurosci (2006) 13(1):60–72. 10.1016/j.jocn.2005.02.013 16410199

[54] Farthing’ JP Krentz’ JR Magnus’ CRA Barss’ TS Lanovaz’ JL Borowsky R Changes in Functional Magnetic Resonance Imaging Cortical Activation with Cross Education to an Immobilized Limb. Med Sci Sports Exerc (2011) 43:1394–405. 10.1249/MSS.0b013e318210783c 21266927

[55] Malone IB Leung KK Clegg S Barnes J Whitwell JL Ashburner J Accurate automatic estimation of total intracranial volume: A nuisance variable with less nuisance. NeuroImage (2015) 104:366–72. 10.1016/j.neuroimage.2014.09.034 PMC4265726 25255942

[56] Japee S Holiday K Satyshur MD Mukai I Ungerleider LG A role of right middle frontal gyrus in reorienting of attention: a case study. Front Syst Neurosci (2015) 9:1–16. 10.3389/fnsys.2015.00023/abstract 25784862 PMC4347607

[57] Ho M-C Huang G-J Tyan Y-S Tseng H-C Weng J-C Neural response to betel quid cues in chewers: a functional magnetic resonance imaging study. Brain Imaging Behav (2018) 13:1135–45. 10.1007/s11682-018-9933-x 30051327

[58] Barbey AK Koenigs M Grafman J Dorsolateral prefrontal contributions to human working memory. Cortex (2013) May 49(5):1195–205. 10.1016/j.cortex.2012.05.022 PMC3495093 22789779

[59] Blumenfeld RS Parks CM Yonelinas AP Ranganath C Putting the Pieces Together: The Role of Dorsolateral Prefrontal Cortex in Relational Memory Encoding. J Cogn Neurosci (2011) Jan 23(1):257–65. 10.1162/jocn.2010.21459 PMC3970078 20146616

[60] Koenigs M Grafman J The functional neuroanatomy of depression: Distinct roles for ventromedial and dorsolateral prefrontal cortex. Behav Brain Res (2009) 201(2):239–43. 10.1016/j.bbr.2009.03.004 PMC2680780 19428640

[61] Bogdanov M Ruff CC Schwabe L Transcranial Stimulation Over the Dorsolateral Prefrontal Cortex Increases the Impact of Past Expenses on Decision-Making. Cereb Cortex (2015) 27:1094–102. 10.1093/cercor/bhv298 26656728

[62] Coutlee CG Huettel SA The functional neuroanatomy of decision making: Prefrontal control of thought and action. Brain Res (2012) 1428:3–12. 10.1016/j.brainres.2011.05.053 21676379 PMC3202063

[63] Skinner MD Aubin H-J Craving’s place in addiction theory: Contributions of the major models. Neurosci Biobehav Rev (2010) 34(4):606–23. 10.1016/j.neubiorev.2009.11.024 19961872

[64] Mitchell DGV The nexus between decision making and emotion regulation: A review of convergent neurocognitive substrates. Behav Brain Res (2011) 217(1):215–31. 10.1016/j.bbr.2010.10.030 21055420

[65] Azizian A Nestor LJ Payer D Monterosso JR Brody AL London ED Smoking Reduces Conflict-Related Anterior Cingulate Activity in Abstinent Cigarette Smokers Performing a Stroop Task. Neuropsychopharmacology (2010) 35(3):775–82. 10.1038/npp.2009.186 PMC2813980 19907418

[66] Salo R Ursu S Buonocore MH Leamon MH Carter C Impaired Prefrontal Cortical Function and Disrupted Adaptive Cognitive Control in Methamphetamine Abusers: A Functional Magnetic Resonance Imaging Study. Biol Psychiatry (2009) 65(8):706–9. 10.1016/j.biopsych.2008.11.026 PMC2678684 19136097

[67] Jacobus J Squeglia LM Meruelo AD Castro N Brumback T Giedd JN Cortical thickness in adolescent marijuana and alcohol users: A three-year prospective study from adolescence to young adulthood. Dev Cogn Neurosci (2015) 16:101–9. 10.1016/j.dcn.2015.04.006 PMC4624050 25953106

[68] Pruessner JC Volumetry of Temporopolar, Perirhinal, Entorhinal and Parahippocampal Cortex from High-resolution MR Images: Considering the Variability of the Collateral Sulcus. Cereb Cortex (2002) 12(12):1342–53. 10.1093/cercor/12.12.1342 12427684

[69] Agster KL Burwell RD Hippocampal and subicular efferents and afferents of the perirhinal, postrhinal, and entorhinal cortices of the rat. Behav Brain Res (2013) 254:50–64. 10.1016/j.bbr.2013.07.005 23872326 PMC3792719

[70] Morrissey MD Takehara-Nishiuchi K Diversity of mnemonic function within the entorhinal cortex: A meta-analysis of rodent behavioral studies. Neurobiol Learn Memory (2014) 115:95–107. 10.1016/j.nlm.2014.08.006 25151400

[71] Schultz W Dopamine signals for reward value and risk: basic and recent data. Behav Brain Functions (2010) 6(1):24. 10.1186/1744-9081-6-24 PMC2876988 20416052

[72] Hyman SE Addiction: A Disease of Learning and Memory. Am J Psychiatry (2005) 9:1414–22. 10.1176/appi.ajp.162.8.1414 16055762

[73] Torregrossa MM Corlett PR Taylor JR Aberrant learning and memory in addiction. Neurobiol Learn Memory (2011) 96(4):609–23. 10.1016/j.nlm.2011.02.014 PMC3138832 21376820

[74] Yuan K Cheng P Dong T Bi Y Xing L Yu D Cortical Thickness Abnormalities in Late Adolescence with Online Gaming Addiction. PloS One (2013) 8(1):e53055. Draganski B, editor. 10.1371/journal.pone.0053055 23326379 PMC3541375

[75] Cavanna AE Trimble MR The precuneus: a review of its functional anatomy and behavioural correlates. Brain (2006) 129(3):564–83. 10.1093/brain/awl004 16399806

[76] Dieter J Hoffmann S Mier D Reinhard I Beutel M Vollstädt-Klein S The role of emotional inhibitory control in specific internet addiction – an fMRI study. Behav Brain Res (2017) 324:1–14. 10.1016/j.bbr.2017.01.046 28174031

[77] Davey J Thompson HE Hallam G Karapanagiotidis T Murphy C De Caso I Exploring the role of the posterior middle temporal gyrus in semantic cognition: Integration of anterior temporal lobe with executive processes. NeuroImage (2016) 137:165–77. 10.1016/j.neuroimage.2016.05.051 PMC4927261 27236083

[78] Hua K Wang T Li C Li S Ma X Li C Abnormal degree centrality in chronic users of codeine-containing cough syrups: A resting-state functional magnetic resonance imaging study. NeuroImage: Clin (2018) 19:775–81. 10.1016/j.nicl.2018.06.003 PMC6031869 29988765

[79] Lessov-Schlaggar CN Pang Z Swan GE Guo Q Wang S Cao W Heritability of cigarette smoking and alcohol use in Chinese male twins: the Qingdao twin registry. Int J Epidemiol (2006) 35(5):1278–85. 10.1093/ije/dyl148 16847025

[80] Liao Y Chen X Tang J Differences of cigarette smoking and alcohol consumption by sex and region in China: a population-based, multi-stage, cluster sampling survey. Lancet (2017) 390:S54. 10.1016/S0140-6736(17)33192-6

